# Antimicrobial Properties of the Polyaniline Composites against *Pseudomonas aeruginosa* and *Klebsiella pneumoniae*

**DOI:** 10.3390/jfb11030059

**Published:** 2020-08-19

**Authors:** Moorthy Maruthapandi, Arumugam Saravanan, John H. T. Luong, Aharon Gedanken

**Affiliations:** 1 Department of Chemistry, Bar-Ilan Institute for Nanotechnology and Advanced Materials, Bar-Ilan University, Ramat-Gan 52900, Israel; lewismartin.jesus@gmail.com (M.M.); saran.bc94@gmail.com (A.S.); 2 School of Chemistry, University College Cork, Cork T12 YN60, Ireland; luongprof@gmail.com

**Keywords:** antimicrobials, polyaniline (PANI), CuO, TiO_2_, SiO_2_, ultrasonication, carbon dots, PANI-composites, *Pseudomonas aeruginosa*, *Klebsiella pneumoniae*

## Abstract

CuO, TiO_2,_ or SiO_2_ was decorated on polyaniline (PANI) by a sonochemical method, and their antimicrobial properties were investigated for two common Gram-negative pathogens: *Pseudomonas aeruginosa* (PA) and *Klebsiella pneumoniae* (KP). Without PANI, CuO, TiO_2_, or SiO_2_ with a concentration of 220 µg/mL exhibited no antimicrobial activities. In contrast, PANI-CuO and PANI-TiO_2_ (1 mg/mL, each) completely suppressed the PA growth after 6 h of exposure, compared to 12 h for the PANI-SiO_2_ at the same concentration. The damage caused by PANI-SiO_2_ to KP was less effective, compared to that of PANI-TiO_2_ with the eradication time of 12 h versus 6 h, respectively. This bacterium was not affected by PANI-CuO. All the composites bind tightly to the negative groups of bacteria cell walls to compromise their regular activities, leading to the damage of the cell wall envelope and eventual cell lysis.

## 1. Introduction

Many pathogens often live for considerable periods on inert surfaces to trigger the transmission of infectious diseases, especially in nursing homes, hospitals, and clinical settings, known as nosocomial or healthcare-acquired infections. The combined use of disinfectants and bactericidal surface coatings could drastically decrease the prevalence of bacterial infections. The surfaces of medical devices or objects in common clinical/hospital areas can be coated with antimicrobial materials because they are used or touched almost daily by health care workers. The prevention of microbial contamination is also critical in the food and pharmaceutical industries, a growing global concern. PA, the most common Gram-negative pathogen, causes hospital-related pneumonia in the United States [1]. This facultative anaerobe is well adapted to proliferate in the absence of oxygen using nitrate or nitrite as a terminal electron acceptor [2,3,4]. Carbapenem-resistant PA is a critical (priority 1) pathogen that infects immunocompromised patients [5] and is linked to ventilator-associated pneumonia and sepsis syndromes. Its adaptable genome also allows PA to attain resistance to different antibiotics [6] and infects the airway, urinary tract, burns, and wounds of immunodeficient patients [7]. KP is the commonest cause of hospital-acquired pneumonia in the United States [8] and pneumonia for inpatient populations. In general, Gram-negative bacteria are more difficult to kill and develop resistance more quickly to antibiotics, compared to Gram-positive counterparts.

Copper occurs as solid metal Cu (0); Cu (I) cuprous ion; Cu (II) cupric ion; and, rarely, Cu (III) [9], and its antimicrobial property was dated back in ancient times [10]. Cu and its alloys serve as antimicrobial agents for both medical and non-medical purposes [11]. In the presence of air, copper is oxidized to CuO, which can be light-activated at ~720 nm [12] to form radicals that impair the bacterial cell wall. TiO_2_ powder or its thin film is stable and photosensitive [13]. TiO_2_ surfaces can become antimicrobial materials due to their abundance and non-toxicity. Illuminations of TiO_2_ induce the release of several reactive oxygen species, and one of the species is hydroxyl radicals (·OH), which is reactive and causing oxidative damage to cellular components, leading to eventual cell lysis. With a bandgap of 3.2 eV [14], the antimicrobial activity of TiO_2_ is attained under UV radiation at <385 nm.

Polyaniline (PANI) has received significant interest in diversified applications because of its low cost, biocompatibility, stability, and low toxicity [15,16,17]. However, PANI materials have some disadvantages, including weak processability and poor mechanical stability [18,19]. Several attempts have been advocated to prepare PANI composite materials with improved activity, physical, and structural properties [20,21,22,23]. Metal oxides can bind the polymer chain in the PANI backbone to strengthen polymer stability [23,24,25] for various pertinent uses [18,25,26,27,28].

As a part of our on-going activities, this paper extends the applicability of the PANI composite with CuO, TiO_2_, or SiO_2_, respectively, against two-Gram negative pathogens: *P. aeruginosa* and *K. pneumoniae*. All Gram-negative bacteria have thin peptidoglycan, which is covered by multiple thin layers of membrane. They are harder to eradicate and quickly develop resistance to antibiotics, compared to Gram-positive bacteria. The polymer composites are characterized by FTIR, XRD, SEM, and zeta potential measurement. A postulated mechanism of the bacterial death is then corroborated and supported by relatable experimental data.

## 2. Experimental Section

### 2.1. Materials

All chemicals of analytical grade were purchased from Sigma–Aldrich Israel and used without any other purification.

#### Synthesis of CDs (Carbon Dots)

CDs were synthesized as described previously [26]. Polyethylene glycol-400 (40 mL) was placed in a glass beaker, which was immersed in an oil bath at 60–70 °C. Sonication was performed by dipping an ultrasonic transducer tip in the liquid. After 2 h of sonication at a 35% amplitude, a yellow color CD solution was attained.

**Preparation of PANI:** The PANI is often synthesized by a chemical or electrochemical oxidative method in an acidic medium with ammonium/potassium persulfates as initiators [29,30,31,32,33]. Simple and green synthesis of conducting polymers is carried out with CDs as an initiator [34,35,36,37]. Resulting stable polymers can also be decorated with various metal oxides by facile ultra-sonication to form polymer-metal oxide composites [38,39,40,41]. In brief, aniline (1.0 g) dissolved in 25 mL of 4M HNO_3_ was admixed with 3 mL of CDs (5 mg). After illumination with UV light for 48 h, the resulting solution became a blackish-brown slurry. The blackish-brown solid was recovered by filtration, followed by extensive washings with distilled water and dried at room temperature.

**Synthesis of PANI-CuO:** As-prepared PANI (100 mg in 90 mL of ethanol) was admixed with copper (II) acetate (20.1 mg in 10 mL of deionized water, 0.012 M). The reaction mixture was sonicated until the temperature reached 70 °C. The solution pH of 8.0–9.0 was maintained by adding an ammonia solution as required. When the blue-tinted solution turned blackish brown, it was placed on an ice bath and subject to sonication for 30 min [27,28]. PANI exhibited a zeta potential of +42 mV, as measured by a Malvern Zetasizer Nano-ZS (Malvern Panalytical, Malvern, UK). 

**PANI-TiO_2_:** An identical reaction was performed using 0.1 g of Ti (IV) isopropoxide, instead of 20.1 mg of copper (II) acetate. 

**PANI-SiO_2_:** In this synthesis, 0.1 g of tetraethyl orthosilicate was used instead of 20.1 mg of copper (II) acetate.

### 2.2. Antibacterial Tests

Overnight incubation of *P. aeruginosa* PAO1 and *K*. *pneumoniae* ATCC 700, 603 were carried out at 37 °C in Luria-Bertani (LB) broth. Detailed information on these two bacterial strains was described in Supporting Information. The antibacterial tests were conducted using a mixture of the polymer composite (500 µL) and the bacterial suspension (500 µL). The mixture was incubated at 37 °C with shaking at 200 rpm (AOSI-2 orbital shaker incubator). An aliquot (100 mL) taken at 0, 3, 6, and 12 h was diluted 10-fold in 20% LB medium and plated on the LB agar plates. The plates were dried and incubated for 16 h at 37 °C. Based on the counting of bacterial colonies at appropriate dilutions, the sum of viable bacteria was calculated by the CFU method. The experiments were performed in triplicate. 

### 2.3. Analytical Techniques 

A Transon 27 spectrometer (Bruker, Bremen, Germany) was used to acquire all FTIR spectra of the polymer composites. The crystalline nature of the polymer materials was analyzed using the X-ray diffraction technique by a Bruker AXS D8 Advance diffractometer. All morphologies of the polymer composites were performed using an FEI Magellan 400 L microscope (FEI, Hillsboro, OR, USA). For SEM analysis, a small quantity of the dried powder was placed on a carbon tape, which was attached to a copper strip. The material was coated with gold to improve the conductivity required for SEM imaging. 

## 3. Result and Discussion

In 4M HNO_3_, the condition used to synthesize PANI aniline with a pKa of 4.6 should be protonated entirely to form the anilinium ions. The synthesized PANI consists of protonated –NH_3_^+^ and aromatic –C_6_H_5_ groups. Homogeneous nucleation of PANI was initiated by the rapid mixing of the acidic solution containing C-dots form PANI irregular tubes, ca. 400–500 nm [33]. Carbon dots (CDs) with negative charges displayed electrostatic interactions with the anilinium ions [34]. The zeta potential of PANI is +42 mV as indicated earlier (Figure S1).

### 3.1. Characterization of composites

**Figure 1:**
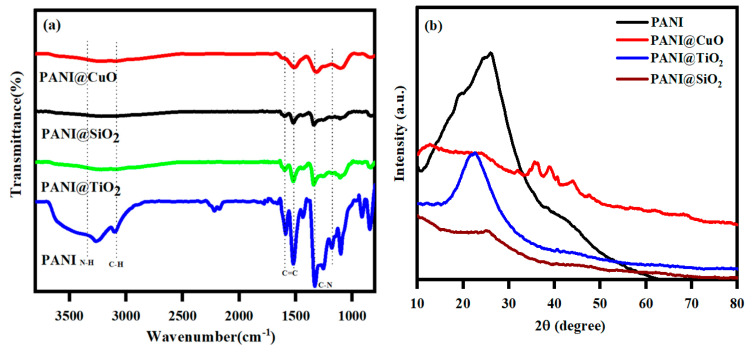
(**a**) FTIR spectra of the polymer composites and (**b**) XRD pattern for the PANI composites of PANI-CuO, PANI-TiO_2_, and PANI-SiO_2_.

FTIR spectra of the polymer composites with major peaks were shown in Figure 1a [35,36,37] and Table 1.

These reduced intensities and disappearances of the peaks confirmed the development of the polymer composites of CuO, TiO_2,_ and SiO_2,_ with PANI. A vast XRD diffraction peak with 2*θ* = 20–40° of PANI could be attributed to the periodicity in the polyaniline structure parallel to the polymer chains (Figure 1b). A broad peak of 2*θ* = 24° was observed for the PANI-SiO_2_ composite, confirming the formation of SiO_2_ on the PANI surface (Figure 1a). This polymer composite also showed a significantly reduced intensity compared to the PANI, indicating the formation of a stable composite between SiO_2_ and the PANI chain. Similar results were also noted for the PANI-TiO_2_ composite with the lowest intensity. Apparently, the PANI and its related PANI-TiO_2_ and PANI-SiO_2_ composite materials were amorphous. The XRD patterns of CuO, TiO_2_, and SiO_2_ were reported elsewhere [38,39,40,41]. The PANI-CuO also exhibited a reduced diffraction intensity around 2*θ* = 24°, and the peaks around 2*θ* = 38–40° are attributed to the CuO. 

### 3.2. Morphology and Particle Size Distribution

**Figure 2:**
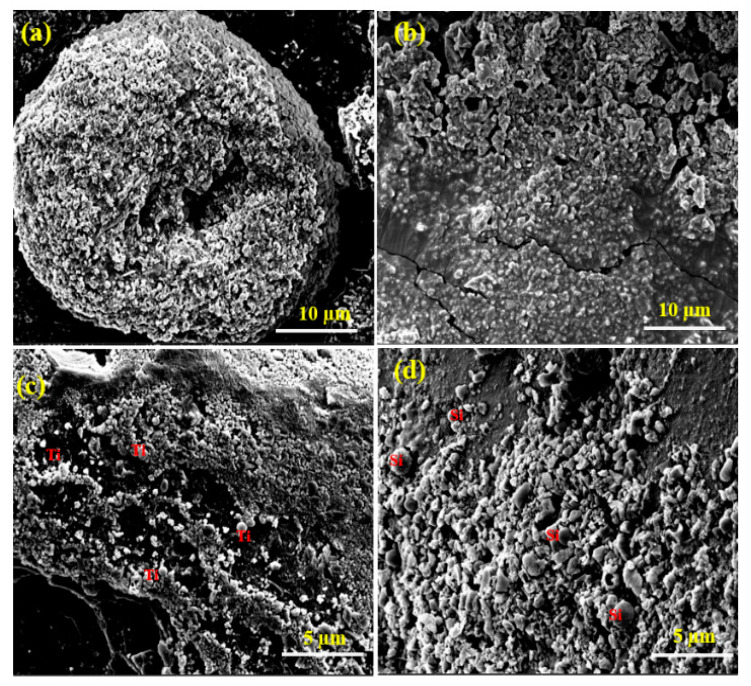
SEM micrographs of PANI (**a**), PANI-CuO (**b**), PANI-TiO_2_ (**c**), and PANI-SiO_2_ (**d**).

As shown in Figure 2, the three PANI composites exhibited irregular morphology with an average diameter of 5 µm. Their corresponding EDX spectra and SEM-EDX elemental analysis spectra confirmed the presence of C, N, O, Au, and Ir, with uniform distribution. For SEM imaging and analysis, the polymer composite material was placed on a carbon tape, which was attached to a copper strip. The anchored composites were then coated with Ir to improve the conductivity required for imaging. Figure 2a,b confirmed the presence of spherical TiO_2_ particles and small flake-shaped SiO_2_ particles with the PANI. The SEM-EDS elemental mapping spectra of the three PANI composites further confirmed the presence of Ti and Si (Figure S2).

**Figure 3:**
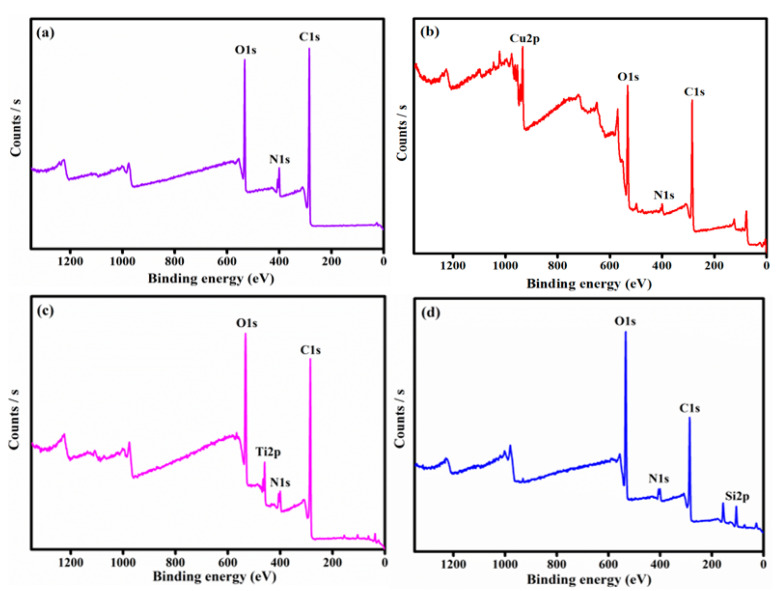
The XPS- spectrum of PANI (**a**), PANI-CuO (**b**), PANI-TiO_2_ (**c**), and PANI-SiO_2_ (**d**).

The presence of C, N, O, and metal ions of Cu, Ti, and Si was observed from the composite XPS spectra as shown in Figure 3. Three new peaks at 838, 123.79, and 886 eV emerged after the adsorption process.

### 3.3. Eradication of P. aeruginosa and K. pneumoniae

There are many literature reports on polymer composites used for eradicating lethal bacteria with metals and metal oxide nanoparticles, such as PANI/polyvinyl alcohol/Ag, PANI@ZnO, PANI/Pt-Pd, PANI/Ag–Pt, PANI-Ag-Au, and Au-PANI -based material [44,45,46,47,48]. However, the synthesis of such polymer- composites is complicated and involves costly noble (Au and Pt) or toxic metals (Ag, and Pd). To avoid the complicated methods and economical cost for the synthesis of PANI composite, the sonochemical method was applied for the formation of highly effective PANI-CuO, PANI-TiO_2,_ and PANI-SiO_2_. The composites were tested against two Gram-negative bacteria as mentioned earlier. For comparison, the effect of PANI (780 µg/mL), CuO, TiO_2,_ or SiO_2_ (220 µg/mL, each) was also investigated as the control experiments.

**Figure 4:**
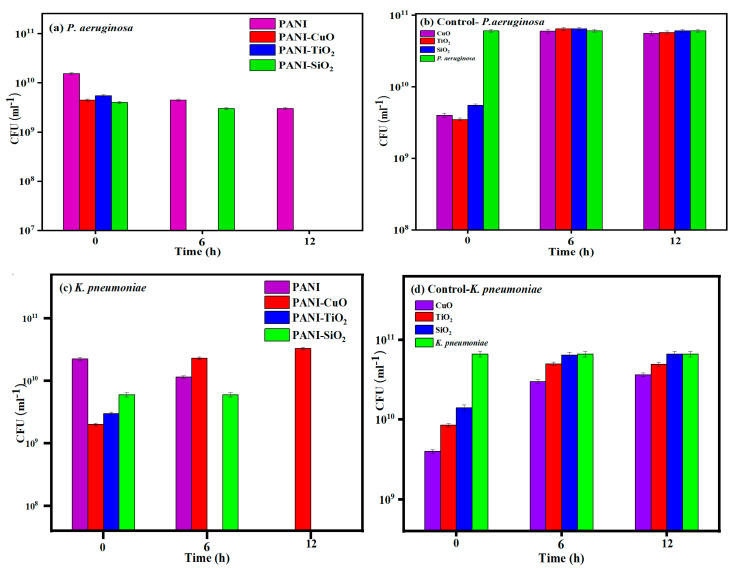
The effect of PANI-CuO, PANI-TiO_2_, and PANI-SiO_2_ on the growth of *Pseudomonas aeruginosa* (PA) (**a**) and *Klebsiella pneumoniae* (KP) (**c**). The effect of CuO, TiO_2_, and SiO_2_ on PA (**b**) and KP (**d**).

Both PANI-CuO and PANI-TiO_2_ completely suppressed the growth of PA after 6 h of its exposure to these two composites. The PANI-SiO_2_ required up to 12 h to eradicate the growth of this bacterium, whereas PANI exhibited a modest antimicrobial effect on PA (Figure 4a). All pure three oxide particles exhibited no antimicrobial activities against PA (Figure 4b). Surprisingly, PANI-CuO showed no antimicrobial activity against KP, whereas PANI-TiO_2_ completely suppressed the growth of KP after 6 h of exposure (Figure 4c). The growth of KP was not affected by PANI-SiO_2_ or PANI during the first 6 h incubation but succumbed after 12 h of exposure (Figure 4d). 

PANI serves as an antimicrobial agent as it exhibits a positive change at pH below 7, which binds to the positively charged membrane of Gram-negative bacteria, consisting of the lipopolysaccharides (LPS). The zeta potential of PA is −29.8 ± 1.5 mV and increases to −25.7 ± 1.4 mV [49] upon its interaction with hexadecyltrimethylammonium bromide (CTAB), a cationic surfactant. Both electrostatic and hydrophobic interactions between CTAB and the bacterial membrane of PA play an important role that leads to cell lysis [49]. In this context, –NH_3_^+^ and –C_6_H_5_ moieties of PANI bind to the bacterial membrane by electrostatic and hydrophobic interactions, respectively. The hydrophobic domain of PANI, –C_6_H_5_, effectively induces membrane disruption or permeabilization due to its interaction with the membrane hydrophobic core [50]. The binding event compromises the bacterial activities due to the leakage of cellular components, and the breakdown of membrane potential, leading to eventual cell lysis. As a cited example, PANI up to 0.5% (w/v) or 5 mg/mL is needed to subdue the growth and replication of *S. aureus* and *E. coli* and [51]. PANI induces the release of hydrogen peroxide, promoting the liberation of hydroxyl radicals to oxidize bacterial biomolecules, leading to cell lysis. *E. coli* [52] is equipped with a defensive enzyme, catalase, which scavenges hydrogen peroxide to benign water and oxygen. Thus, high PANI concentrations are necessary to promote the H_2_O_2_ level beyond the scavenging threshold of this enzyme. The PANI concentration used in this study was 0.78 mg/mL, which was significantly below 5 mg/mL, the level needed to eradicate some bacteria as mentioned previously. Nevertheless, this polymer was effective against the growth of KP after 12 h of its exposure but exhibited a very modest effect on the growth of PA.

Without the polymer, both PA and KP were not affected by CuO, TiO_2,_ and SiO_2,_ at 220 µg/mL. Several bacteria, including PA and KP, are only suppressed by nano-SiO_2_ at concentrations above 0.625 μg/mL [53].

The multidrug-resistant PA [19] can express efflux pumps, β-lactamases, and impermeable outer membrane proteins to overcome the effect of copper ions. The effective Cu concentration against bacteria also depends upon bacterial growth medium compositions and time of exposure. As an example, the minimum inhibitory concentration for CuSO_4_ copper sulfate for several strains of *E. coli* ranges between 16 and 20 mM (1017–1271 µg/mL) [54]. Both TiO_2_ and CuO could bind to the negatively charged bacterial membrane at pH 5 used in the microbial tests. The overall negative charge of two Gram-negative cell walls is attributed to the presence of considerable carboxylic external groups located in bacterial walls. The zeta potential of TiO_2_ nanoparticles is 31 ± 0.5 mV at pH 3.0 and approaches 0 at pH = 5.8 ± 0.1 [55]. The surface of the CuO nanoparticles is positively charged between pH 2 and 6 (44.2 ± 1.1 and 36.2 ± 0.9 mV), and approaches neutral at elevated pH 10 [56].

The decoration of CuO, TiO_2,_ and SiO_2_ on PANI should also have some additional effects on the two Gram-negative bacteria, as their cell walls encompass lipids and polysaccharides with low rigidity and strength. The lipopolysaccharides with negative charges are attracted to such oxides with opposite charges. This electrostatic interaction impairs the syntheses of the cell wall, nucleic acids, and proteins, which are required for bacterial growth and replication. The cell membrane is eventually ruptured to release cytoplasmic contents, leading to cell lysis. There was a synergistic effect between PANI and the three oxides, towards the eradication of the two tested Gram-negative bacteria. However, it is not clear whether there is any appreciable amount of reactive oxygen species generated from the polymer composites under ambient light. The bandgap (energy gap) of CuO and TiO_2_ is 1.7 and 3.2 eV, respectively, compared to 9 eV for SiO_2_ [57]. Thus, CuO can be activated by light with a wavelength of < approx. 720 nm, compared to UV for TiO_2_. All the pristine oxides—TiO_2_, CuO, and SiO_2_—exhibited no antimicrobial activities against PA and KP, as the experiments were simply conducted without light illumination, implying no significant ROS were released under this experimental condition. PANI generates π−π* transition under visible light irradiation to transport the exciting electrons into the TiO_2_ conduction. The electrons can be moved to an adsorbed electron acceptor to yield oxygenous radicals [58]. The mechanism for the formation of metal oxides by sonochemistry was reported by our group [59,60]. 

This study focuses on two Gram-negative bacteria, but the synthesized polymer composites can be extended to Gram-positive bacteria. Gram-negative bacteria with a two-membrane barrier are more resistant to antibodies and antibiotics than Gram-positive counterparts [61]. The antimicrobial mechanism of antibiotics generally falls within one of four mechanisms: (i) inhibition or regulation of enzymes involved in cell wall synthesis, (ii) interfering nucleic acid metabolism and repair, (iii) inhibition of protein synthesis, and (iv) disruption of membrane structure [62]. Emerged multidrug-resistant Gram-negative pathogens pose a significant challenge in the development of new antibiotics and antimicrobial agents. The generation of ROS by metal nanoparticles could be one vital route to overcome the limitation of membrane penetration to kill bacteria. These radicals cause the damage of lipids and proteins and oxidize the deoxynucleotide pool. As mentioned earlier, antibiotics with specific mechanisms of action have been advocated; however, this classical view should be expanded to include the role of ROS generated by metal nanoparticles and polymer composites for bacterial eradication. 

A brief discussion was noted here for the toxicity of copper, TiO_2,_ and SiO_2_. Indeed, copper is an essential element for all living organisms, as an adult body contains about 1.2–1.4 mg of copper/kg of body mass. A human can uptake one mg of copper daily, and extra amounts of copper are released in bile and discharged in feces [63]. There is no significant cytotoxicity/inhibition when Chinese hamster lung fibroblast V79 cells are exposed up to 400 ppm of P25 (5.9 nm, 99% anatase), MTI5 (34.1 nm, 80% anatase, and 20% rutile) and bulk TiO_2_. TiO_2_ nanoparticles (NPs) are aggregated under UV illumination at 254 nm or 365 nm with diminishing photocatalytic activities and exhibit no significant effect on cytotoxicity [64]. The TiO_2_ level used in this work was only 220 µg/mL or 220 ppm, below the plausible toxicity level of TiO_2_, 400 ppm. The inhibition concentration (IC_50_) of SiO_2_ against U373MG cells U373MG (human glioblastoma cell line) at 24 h has been reported [65]. This value is dependent on the size and surface charges, ranging from 680–6380 µg/mL. Again, such cytotoxic levels are significantly higher than 220 µg/mL used in this work. Based on its non-toxicity, TiO_2_ has been formulated in a plethora of industrial and consumer products, including sunscreen lotion, toothpaste, and soap. The U.S. Food and Drug Administration (FDA) has confirmed the safety aspect of TiO_2_ in cosmetics, including products intended for use around the eye [66]. Nevertheless, cytotoxicity of any material is concentration-dependent, and its effect is different for different cell lines. Besides various conventional techniques for probing cytotoxicity of materials/nanomaterials, cell-based impedance spectroscopy with multiplexing can be used to probing the cytotoxicity effect of antimicrobial agents for various cell lines. The impedance can be monitored continuously to reflect cell attachment and spreading on a confined electrode surface. The impedance will change drastically when adhered cells are exposed to toxic chemicals and nanomaterials, which impair their attachment and other cell behavior [67,68,69]. This simple and precise technique provides quantitative data related to cytotoxicity/cytosis for in vitro cell systems in the presence of plausible toxic compounds. 

## 4. Conclusions

CuO, TiO_2_, or SiO_2_ has no antimicrobial effect against PA and KP. Each PANI composite, however, shows a different antimicrobial activity towards these two Gram-negative bacteria. Both electrostatic and hydrophobic interactions of the PANI composites with the bacterial membrane compromise cell activities, adversely affecting their growth and proliferation. This mechanism is similar to the effect of surfactants on several microorganisms [49,70,71,72]. PANI also induces the formation of hydrogen peroxide, leading to the production of hydroxyl radicals to oxidize bacterial biomolecules. The release of the above ROS damages bacterial active enzymes, DNA, and proteins that sustain cell proliferation and other important activities [73,74]. The superoxide radical with a negative charge is repulsed by the bacterial membrane with the same charge; therefore, it only acts on the outer surface of bacteria. In contrast, neutral hydrogen peroxide should be able to penetrate the bacterial cell wall to trigger cell lysis. 

The use of the polymer composites to prevent the growth of PA is an important finding considering its biofilm formation [75,76], which exhibits intrinsic resistance to different types of chemotherapeutic agents and antibiotics. PA is one of the prime causes of morbidity and mortality in ~80% of patients with cystic fibrosis [77,78]. It is also a common cause of pneumonia infections in intensive care units [79]. PA can form multicellular aggregates or biofilms, which act as a direct barrier to phagocytic cells and antibiotics [80,81]. The application of polymer composites can be extended to a variety of other applications such as food packaging, textile, coating, military, and household equipment. In practice, the polymer composites can be used in combination to eradicate a mixture of several bacteria. It is paramount important to access the antimicrobial properties against some common multi-drug-resistant bacteria, including foodborne pathogens. Besides *Pseudomonas,* two emerging pathogenic Gram-negative *Enterobacteriaceae* and *Acinetobacter* species are resistant to most available antibiotics [82]. The assessment of the synthesized PANI composites against such pathogens is an important aspect of future endeavors. Of course, other biopolymers and electropolymerized polymers including polypyrrolepropylic acid [83] and poly(*N*-acetyltyramine) [84] could be used to form polymer composites with metal nanoparticles towards the development of a new class of antimicrobial agents.

## Figures and Tables

**Table 1:** FTIR peaks and peak assignments of polyaniline (PANI) and its composites.

Materials	Major Peaks and Assignments
PANI	The broad peak at 3272 cm^−1^, N–H stretching.
	The peak at 3098 cm^−1^, C–H aromatic stretching vibration.
	The peaks at 1595 and 1516 cm^−1^, C=C stretching of quinoid and benzenoid rings, respectively.
	The peak at 1331 cm^−1^, C–N stretching of the secondary aromatic amines.
	The peaks at 1176 and 1252 cm^−1^, =C–H in-plane vibrations.
	The peak at 825 cm^−1^, out-of-plane bending of C–H in the 1,4-disubstituted benzene ring.
	The broadband ~1059–1724 cm^−1^
TiO_2_ nanoparticles	The broadest one at 3500 cm^−1^, stretching vibration of the hydroxyl group.
	The second band ~1630 cm^−1^, bending mode of water Ti–OH.
	The prominent peak at 1383 cm^−1^, Ti–O modes [42,43].
PANI-TiO_2_ and PANI-SiO_2_	The amine stretching vibrations of N–H and C–N stretching are extremely reduced sharp narrow peaks. The aromatic stretching vibration ~3098 cm^−1^ and 1252–1085 cm^−1^ (=C–H in-plane vibration) entirely disappeared due to the strong formation of metal oxides onto the polymer [38].

## Supplementary Materials

The following are available online at https://www.mdpi.com/2079-4983/11/3/59/s1, Figure S1: The zeta potential of PANI, Figure S2: SEM-EDS elemental mapping spectra of PANI-CuO, PANI-TiO_2_, and PANI-SiO_2_.
