# Supplementary material for: Antimicrobial Properties of the Polyaniline Composites against Pseudomonas aeruginosa and Klebsiella pneumoniae
Source: J Funct Biomater. 2020 Aug 19;11(3):59. doi: 10.3390/jfb11030059 (PMC7566003; doi:10.3390/jfb11030059)
Supplement: Supplementary file 1 [file jfb-11-00059-s001.pdf]

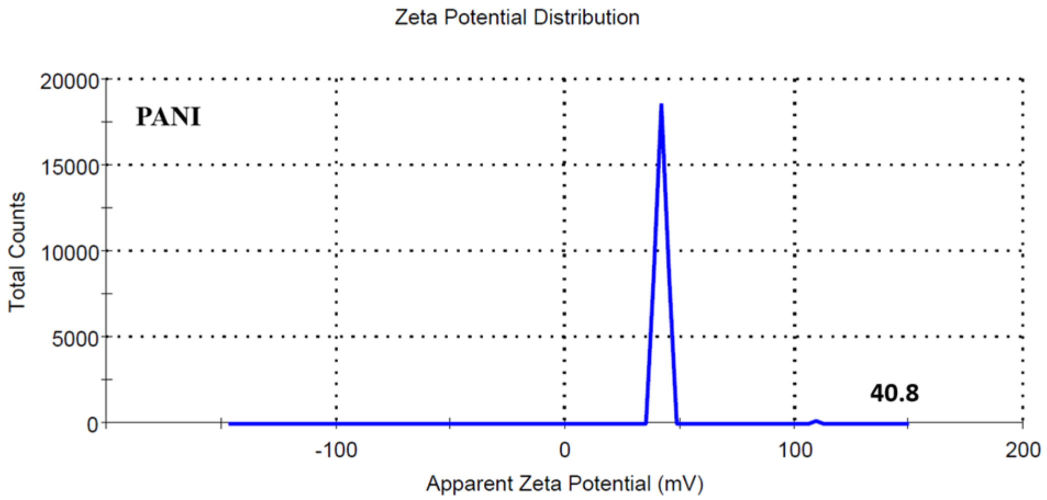

Figure S1. Zeta potential of PANI was measured to be about 40.8 mV.

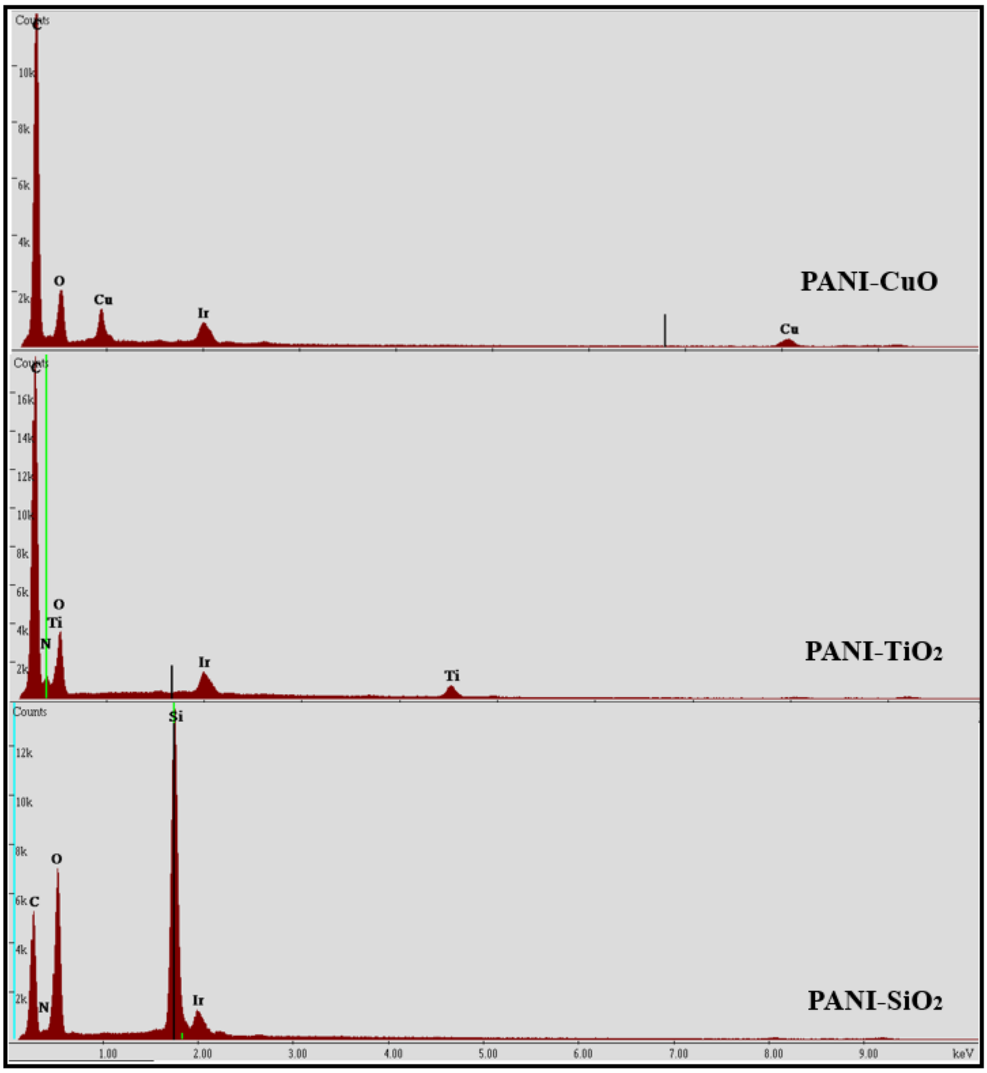

Figure S2. SEM-EDS elemental mapping spectra of the PANI-CuO, PANI-TiO_2, and PANI-SiO_2.

Bacterial strains. *Klebsiella pneumoniae*, ATCC® 700603™ is used as Clinical Laboratory Standards Institute (CLSI) and European Committee on Antimicrobial Susceptibility Testing (EUCAST) positive control for extended-spectrum beta-lactamase ESBL tests [1]. The bacterium is designed as Biosafety Level 2

Pseudomonas aeruginosa PAO1 is a derivative of the original Australian PAO isolate. It is the most commonly used strain as its complete genome sequence is known [2]. It has been distributed worldwide to laboratories and strain collections [3].

References

1. https://www.atcc.org/search#q=K.%20pneumoniae%20ATCC%20700603&sort=relevancy
2. Stover. C.K.; Pham, X.Q.; Erwin, A.L.; Mizoguchi, S.D.; Warrener, P; Hickey M.J.; Brinkman, F.S.L.; Hufnagle, W.O.; Kowalik, D.J.; Lagrou, M.; et al. Complete genome sequence of Pseudomonas aeruginosa PAO1, an opportunistic pathogen. *Nature* **2000**, *406*, 959–964.
3. https://www.pseudomonas.com/strain/show/107.
